# Trend clustering from COVID-19 tweets using graphical lasso-guided iterative principal component analysis

**DOI:** 10.1038/s41598-022-09651-6

**Published:** 2022-04-05

**Authors:** Ryosuke Harakawa, Tsutomu Ito, Masahiro Iwahashi

**Affiliations:** Department of Electrical, Electronics and Information Engineering, Nagaoka University of Technology, Nagaoka, 940-2188 Japan

**Keywords:** Computer science, Information technology, Data mining

## Abstract

This article presents a method for trend clustering from tweets about coronavirus disease (COVID-19) to help us objectively review the past and make decisions about future countermeasures. We aim to avoid detecting usual trends based on seasonal events while detecting essential trends caused by the influence of COVID-19. To this aim, we regard daily changes in the frequencies of each word in tweets as time series signals and define time series signals with single peaks as target trends. To successfully cluster the target trends, we propose graphical lasso-guided iterative principal component analysis (GLIPCA). GLIPCA enables us to remove trends with indirect correlations generated by other essential trends. Moreover, GLIPCA overcomes the difficulty in the quantitative evaluation of the accuracy of trend clustering. Thus, GLIPCA’s parameters are easier to determine than those of other clustering methods. We conducted experiments using Japanese tweets about COVID-19 from March 8, 2020, to May 7, 2020. The results show that GLIPCA successfully distinguished trends before and after the declaration of a state of emergency on April 7, 2020. In addition, the results reveal the international argument about whether the Tokyo 2020 Summer Olympics should be held. The results suggest the tremendous social impact of the words and actions of Japanese celebrities. Furthermore, the results suggest that people’s attention moved from worry and fear of an unknown novel pneumonia to the need for medical care and a new lifestyle as well as the scientific characteristics of COVID-19.

## Introduction

The outbreak of coronavirus disease (COVID-19) has seriously affected human health and economic activity around the world. Twitter (https://twitter.com) has become an important source of information^1–3^, and various *tweets* (i.e., short text messages) have been disseminated widely^4–6^. Detecting trends in daily tweets allows us to objectively look back over the past based on data. This helps us make decisions about future countermeasures.

From the perspective of computational social science, the analysis of trends in Twitter has been studied. Some literature^7–9^ has visualized observations such as news and public sentiment about COVID-19 over time. Karami et al.^10^ used the valence aware dictionary and sentiment reasoner^11^, which is a sentiment analyzer in natural language processing. They observed monthly changes in the sentiment of tweets regarding COVID-19 vaccines. Yang and Chen^12^ investigated the relationship between sentiment and the increase in infections using sentiment analysis for tweets.

In contrast to the above studies, the studies^13–17^ focused on the topic analysis of tweets. Taneja et al.^13^ analyzed activity trends on Twitter and suggested its potential as a vehicle for disseminating scientific information during the pandemic. Caldera et al.^14^ forecasted trends in hate speech in Sri Lanka using the Box–Jenkins method^18^. Arpaci et al.^15^ detected burst topics from the public attention paid to the COVID-19 epidemic using evolutionary clustering^19^. Karmakar and Das^16^ proposed a Bayesian estimation model and showed an upward trend in cyberbullying-related tweets since mid-March 2020. Latent Dirichlet allocation^20^, which is a well-known topic modeling technique, was used in the study^21^. The study^21^ found hidden topics using tweets and determined trending topics based on the number of tweets for each topic. Furthermore, dynamic topic modeling^17^ was proposed for analyzing the COVID-19 Twitter narrative among the United States governors and presidential cabinet members. This method tracked the evolution of sub-topics related to risk, testing, and treatment.

Unlike the above related work, this study aims to cluster trends to gain an overview of daily changes in many topics. One of our contributions is to present the novel research task of trend clustering. In the proposed method, we regard daily changes in the frequencies of each word in tweets as time series signals. The proposed method also defines time series signals with single peaks as target trends. This is because we aim to detect not the usual trends based on seasonal events but essential trends caused by the influence of COVID-19. Importantly, we remove trends with indirect correlations because they are generated by other essential trends. Based on this unique idea, we consider our aim to be to find trends with similar waveforms.

In this article, we propose graphical lasso-guided iterative principal component analysis (GLIPCA). First, we construct a partial correlation network that connects only trends with direct correlations using the graphical lasso algorithm^22^. This processing enables us to select essential trends that form significant clusters from many trends. Simultaneously, this processing models essential trends using unimodal Gaussian distributions with direct correlations. Thus, in this study, essential trends are defined as time series signals with direct correlations, whose wave forms are similar to unimodal Gaussian distributions. In other words, we can evaluate the applicability of the proposed method. Clustering trends generated from other distributions is out of scope of this work. Second, we formulate trend clustering as the detection of dense nodes in the partial correlation network. Specifically, we improve the hyperlink-induced topic search (HITS) algorithm^23^, which is equivalent to principal component analysis (PCA) for a network structure^24^. The proposed method iteratively extracts the first principal component and reconstructs the network so that the corresponding nodes can be removed. This iterative processing including the network reconstruction is novel compared with the HITS algorithm that extracts orthogonal bases from the given network. Thus, unlike other clustering methods, our method does not duplicate members in different clusters, and the number of clusters is automatically determined. These advantages overcome the difficulty of quantitative evaluating the accuracy of trend clustering. Specifically, we can determine the most accurate results by monitoring the modularity^25^, i.e., the quality measure of community detection in a complex network. In the subsequent sections, we present the results of experiments using Japanese tweets about COVID-19 from March 8, 2020, to May 7, 2020. The results show that GLIPCA successfully distinguished trends before and after the declaration of a state of emergency on April 7, 2020.

## Results

In this experiment, we used a computer with Intel 3.60 GHz CPU and 32 GB RAM. The OS was Ubuntu 18.04 LTS. All programs were implemented by Python 3.6.7.

**Table 1:** (a) Early trend words and (b) late trend words.

**(a)**	
Joining a company, interest rates, Bakatono (Japanese TV program and its character), the Drifters (Japanese comedian group), Spain,	
Italy, Korea, Kazuko Kurosawa (Japanese comedian), Japan, Hitoshi Matsumoto (Japanese comedian), immigration, interest rate cut,	
Hanshin Tigers (Japanese baseball team), Morisanchu (Japanese comedian group), Tom Hanks, autograph letter, Lorenzo Sanz,	
Inspection, IOC (International Olympic Committee), president, woman, bank of Japan, the dead,	
Shintaro Fujinami (baseball player in Hanshin Tigers), novel pneumonia, positive, Katsuya Maiguma (Japanese actor), player,	
Sense of smell, graduation, returning to one’s country, mourning, virus, Kozo Tashima (Japanese former soccer player),	
Pandemic, Masataka Nashida (Japanese former baseball player), company	
**(b)**	
Mask, ATARASHIICHIZU (Japanese music group), home, Sumo wrestler, TV Asahi corporation, video,	
Kumiko Shiratori (Japanese comedian), Takehiko Orimo (Japanese former basketball player), remaining at home, entering school, now,	
Tamao Akae (Japanese broadcaster), comment, broadcaster, by-election, Remdesivir,	
Hirofumi Yoshimura (governor of Osaka prefecture), Miki Sumiyoshi (Japanese broadcaster), Cologne, therapeutic drug,	
Hanamaru (alias of a Japanese TV program), Takadagawa (name of a Sumo stable), Kotaro Shiga (Japanese actor), gold’s gym,	
Children’s day (May 5th for each year), request, Pachinko (Japanese gambling machine), Mitz Mangrove (Japanese entertainer),	
Leaving the hospital, announcement, Gotoku Sakai (Japanese soccer player), life, support, benefit, Jun-ichi Ishida (Japanese actor),	
Constitutional amendment, golden weak (Japanese holidays during the last week of April up to the first week of May), end,	
Kumiko Okae (Japanese actress), Yuta Tomikawa (Japanese broadcaster), Shinzo Abe (Japanese former prime minister),	
Takashi Okamura (Japanese comedian), self-restraint, Yoshio Tateishi (Japanese businessman), crisis, Mainichi Broadcasting System,	
Person, long holidays, Atsushi Kataoka (Japanese former baseball player), report station (Japanese TV news program),	
Inter-high school competition, restart, donation, crude oil, extension, constitution, business suspension, Baku Owada (Japanese actor)	

In this table, brackets show notations. We show English translations of the original Japanese.

**Figure 1:**
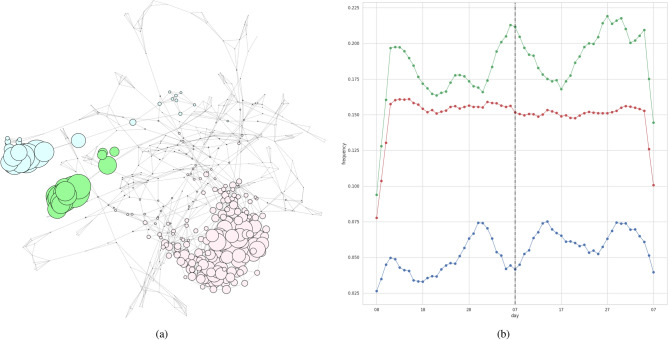
Results for IPCA. The first, second, and third trend clusters are denoted by red, blue, and green, respectively. *B* was set to 6, yielding a *Q* of 0.156. (**a**) The obtained trend clusters. The size of each node is determined by the degree to which the corresponding trend belongs to its clusters. (**b**) Time series signals for each cluster. We show averages of all trends in each cluster. The vertical line indicates the day of the state-of-emergency declaration (April 7, 2020).

**Figure 2:**
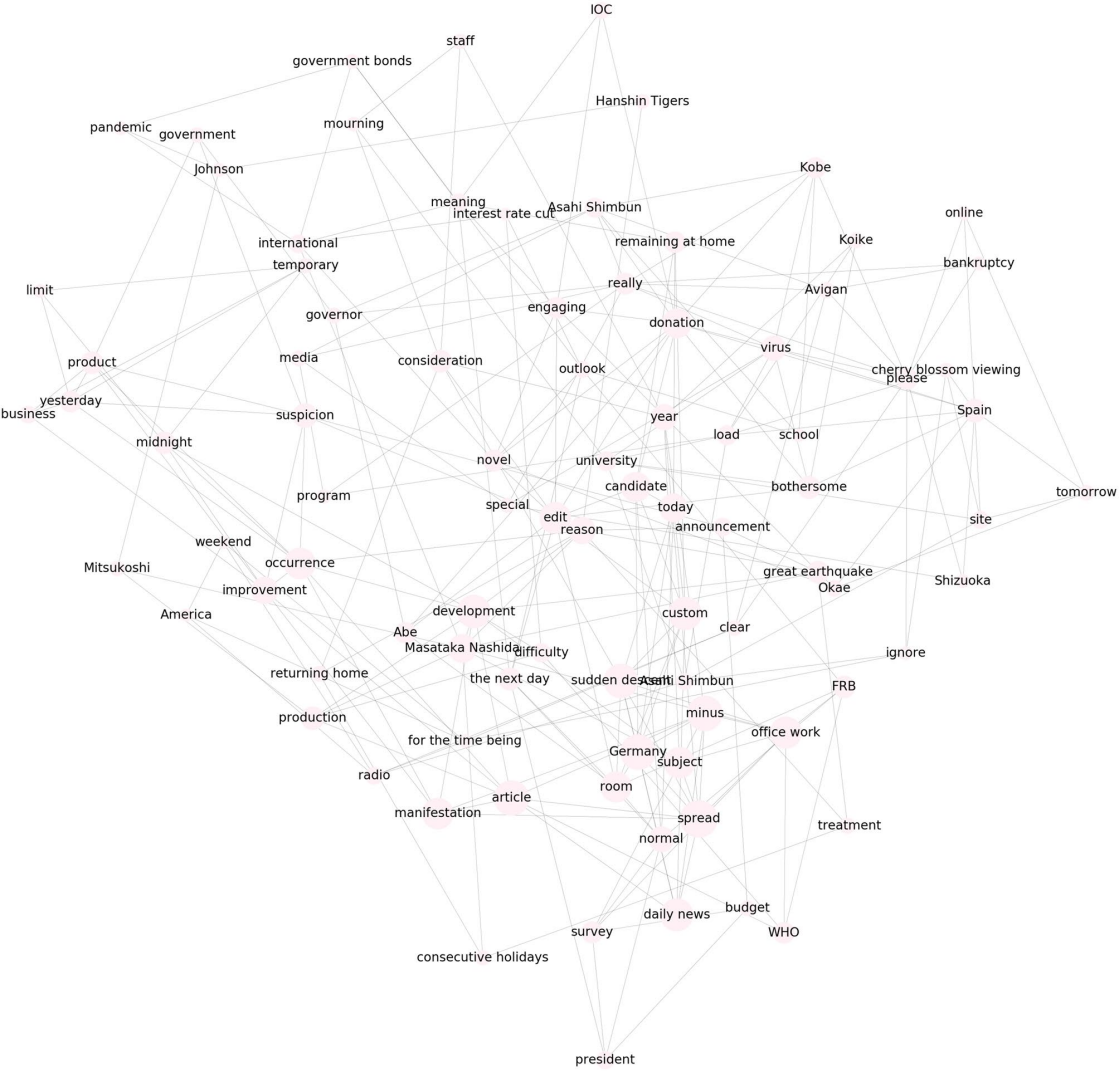
First trend cluster in Fig. 1a. Words corresponding to each trend are shown. For visualization, only 90 words in descending order of the degree to which each trend belongs to the cluster are shown.

**Figure 3:**
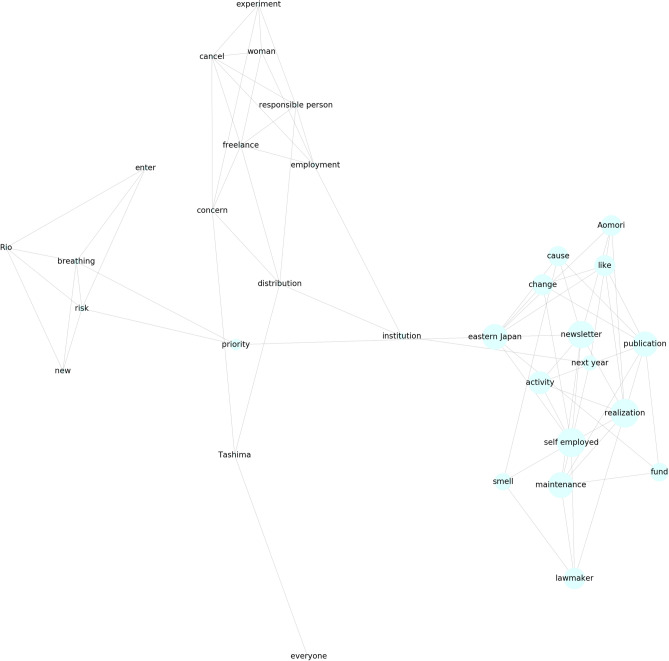
Second trend cluster in Fig. 1a. The words corresponding to each trend are shown.

**Figure 4:**
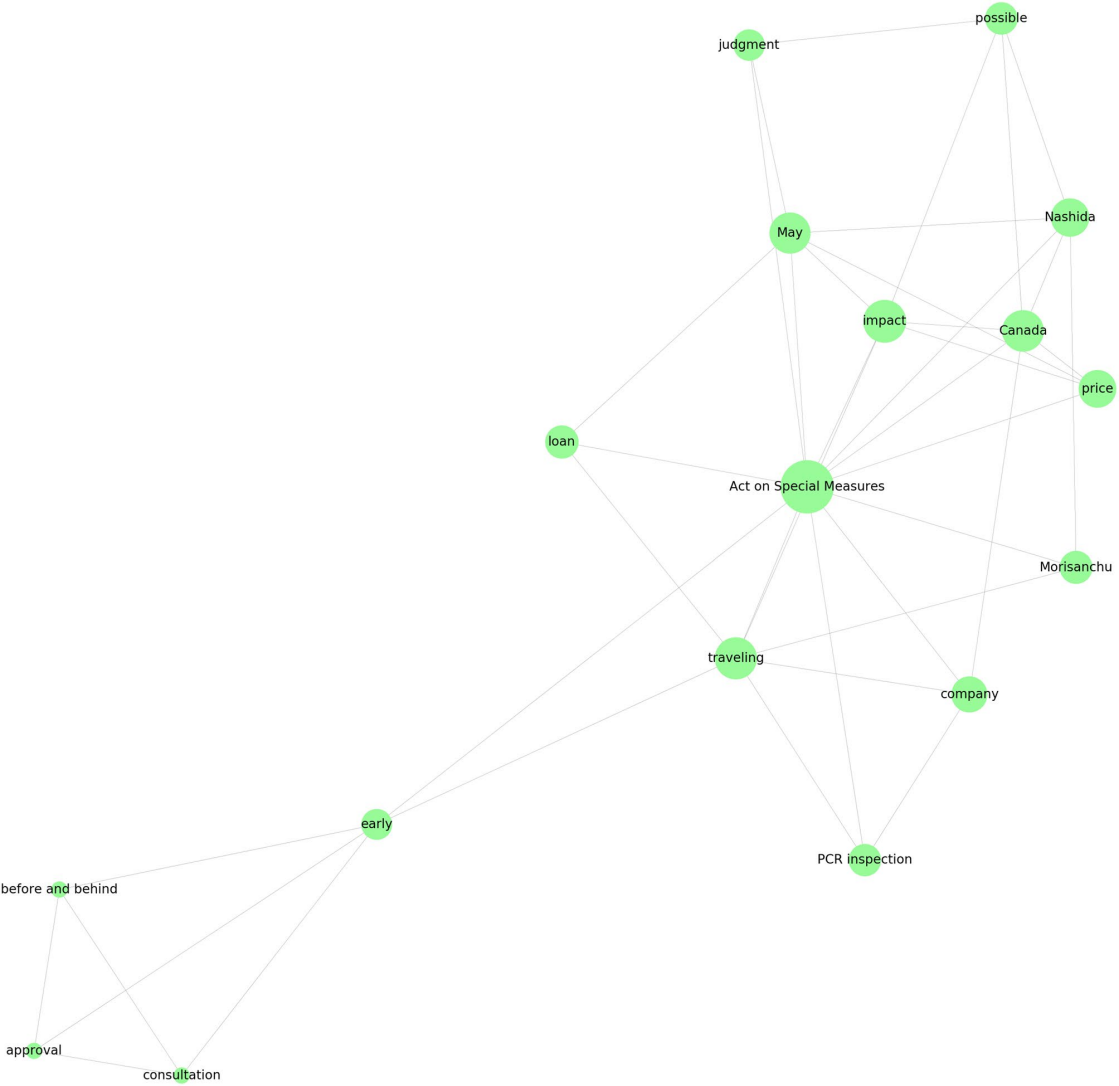
Third trend cluster in Fig. 1a. The words corresponding to each trend are shown.

### Data used in this study

A state of emergency was declared by the Japanese government on April 7, 2020. Tension among the public had increased during this period because such a declaration was the first in Japan. We assume that major trends should be divided into trends before and after the state-of-emergency declaration. To verify this assumption, we crawled Japanese tweets from March 8, 2020, to May 7, 2020 (30 days before and after the state-of-emergency declaration).

For each day during the above period, we collected important words defined in the news analysis about COVID-19^26^ (http://agora.ex.nii.ac.jp/crisis/covid-19/mass-media/word/). Using articles on Yahoo! News (https://news.yahoo.co.jp/), the important words were defined as ones that frequently appeared on the target day but did not frequently appear on other days. From the important words, we collected nouns that only appear on or before (after) April 7, 2020, and refer to them as *early (late) trend words*. Early trend words and late trend words defined in reference to the study^26^ are shown in Table 1.

We defined the Japanese corresponding to “$*$ (corona OR pneumonia)” as the set of queries. Here, $*$ is either an early trend word or late trend word. Note that COVID-19 is commonly called “corona” in Japan. Using Twitter API (https://developer.twitter.com/en/docs/twitter-api), we collected up to 500 tweets for each day and each query from March 8, 2020, to May 7, 2020. Here, we determined the number of 500 because Twitter API can deliver up to 500 tweets per request. As a result, we collected about 7, 736 tweets on average per day and 471, 925 tweets in total during the whole period.

As in the study^27^, we performed pre-processing for each tweet. Concretely, using a natural language processing tool called Janome (https://mocobeta.github.io/janome/en/), we performed morphological analysis^28^ and extracted only the nouns. We then removed stop words as defined in https://www.kaggle.com/lazon282/japanese-stop-words. Moreover, we removed words that consist of only one character because they are likely to be trivial symbols or numbers. Note that Japanese nouns do not change inflection. For example, singular nouns are not distinguished from plural ones. Thus, we did not perform lemmatization^29^.

Furthermore, we regarded words with a small document frequency to be irrelevant to COVID-19. Thus, we removed words whose document frequencies were less than $1\%$ of the total number of tweets from the dataset for each day. The number of the remaining words is denoted by *M*. Subsequently, trend clustering, i.e., an analysis of changes in the daily frequencies of the remaining *M* words, was performed.

### Performance verification

We verify that GLIPCA achieves the following purposes:

**(i)** Does GLIPCA successfully divide major trends into trends before and after the state-of-emergency declaration?

**(ii)** Does GLIPCA model essential trends using unimodal Gaussian distributions with direct correlations?

As a reference method, we implemented IPCA, which is GLIPCA without the graphical lasso algorithm^22^. We set the range of *B* to $\{1, 2, \ldots , 7\}$ and determined the value of *B* that gave the best *Q*. As described in “Methods”, we regard the frequencies of each word as time series signals and applies a moving average filter to the signals for smoothing. A parameter *B* is a width of the moving average filter. Moreover, we quantitatively evaluate the clustering results by modularity *Q*. The modularity is a quality measure, which is generally used for flat community detection in a network. Figures 1, 2, 3 and 4 show results of trend clustering by IPCA. We show an overview of the trend clusters in Fig. 1a. It is difficult to show the trends (words) corresponding to each node while preserving high visibility. Therefore, we separately describe the trends for each cluster in Figs. 2, 3 and 4. Figure 1b shows the time series signals over time for each trend cluster. These figures confirm that IPCA cannot achieve (i). Moreover, we verified whether the obtained trends were generated from unimodal Gaussian distributions. To do this, we used the Shapiro–Wilk test^30^ and present the results in Table 2(a). This confirms that IPCA cannot achieve (ii), suggesting that IPCA may detect the usual trends based on seasonal events. Note that it is clear that IPCA includes indirect correlations because it does not have any functions that remove them.

**Figure 5:**
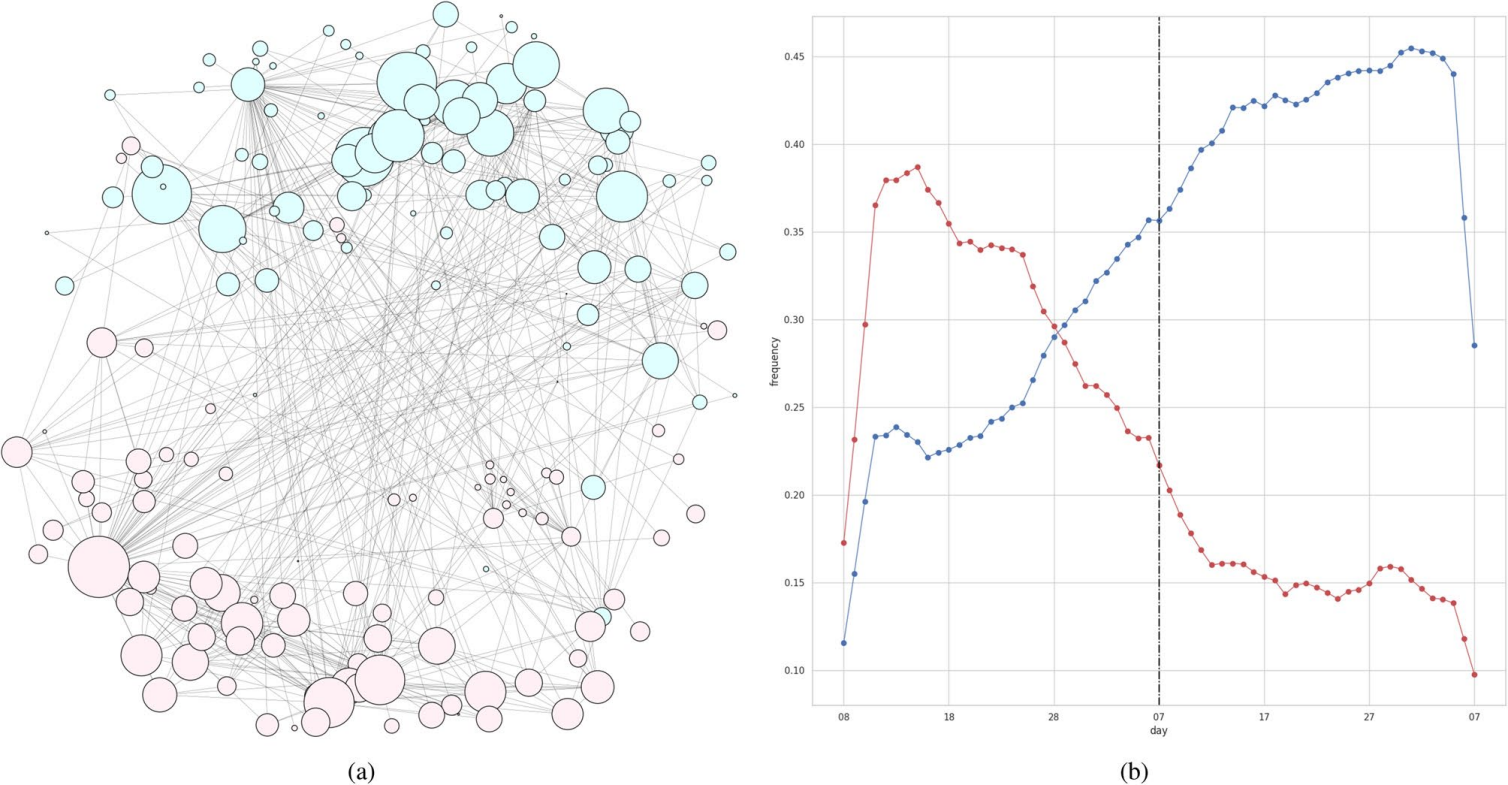
Results for GLIPCA. The first and second trend clusters are denoted by red and blue, respectively. *B* and $\rho$ were set to 6 and 0.015, yielding a *Q* of 0.229. (**a**) The obtained trend clusters. The size of each node is determined by degree to which the corresponding trend belongs to its clusters. (**b**) Time series signals for each cluster. We show the averages of all trends in each cluster. The vertical line indicates the day of the state-of-emergency declaration (April 7, 2020).

**Figure 6:**
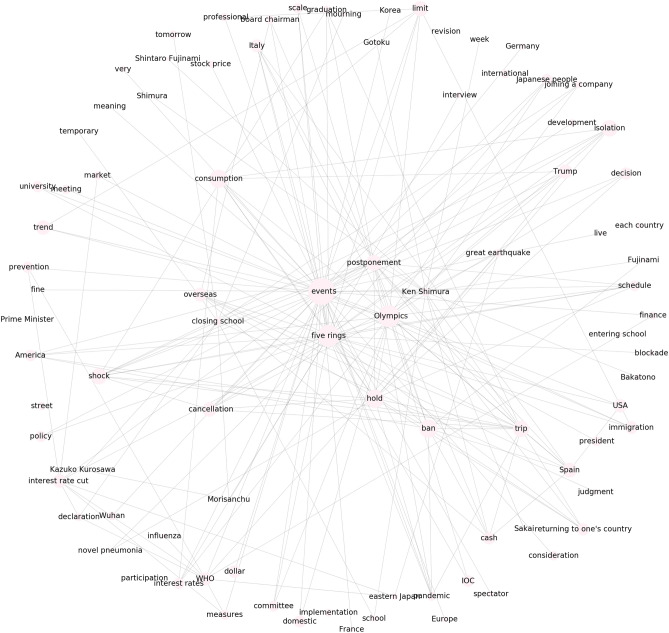
First trend cluster in Fig. 5a. The words corresponding to each trend are shown.

**Figure 7:**
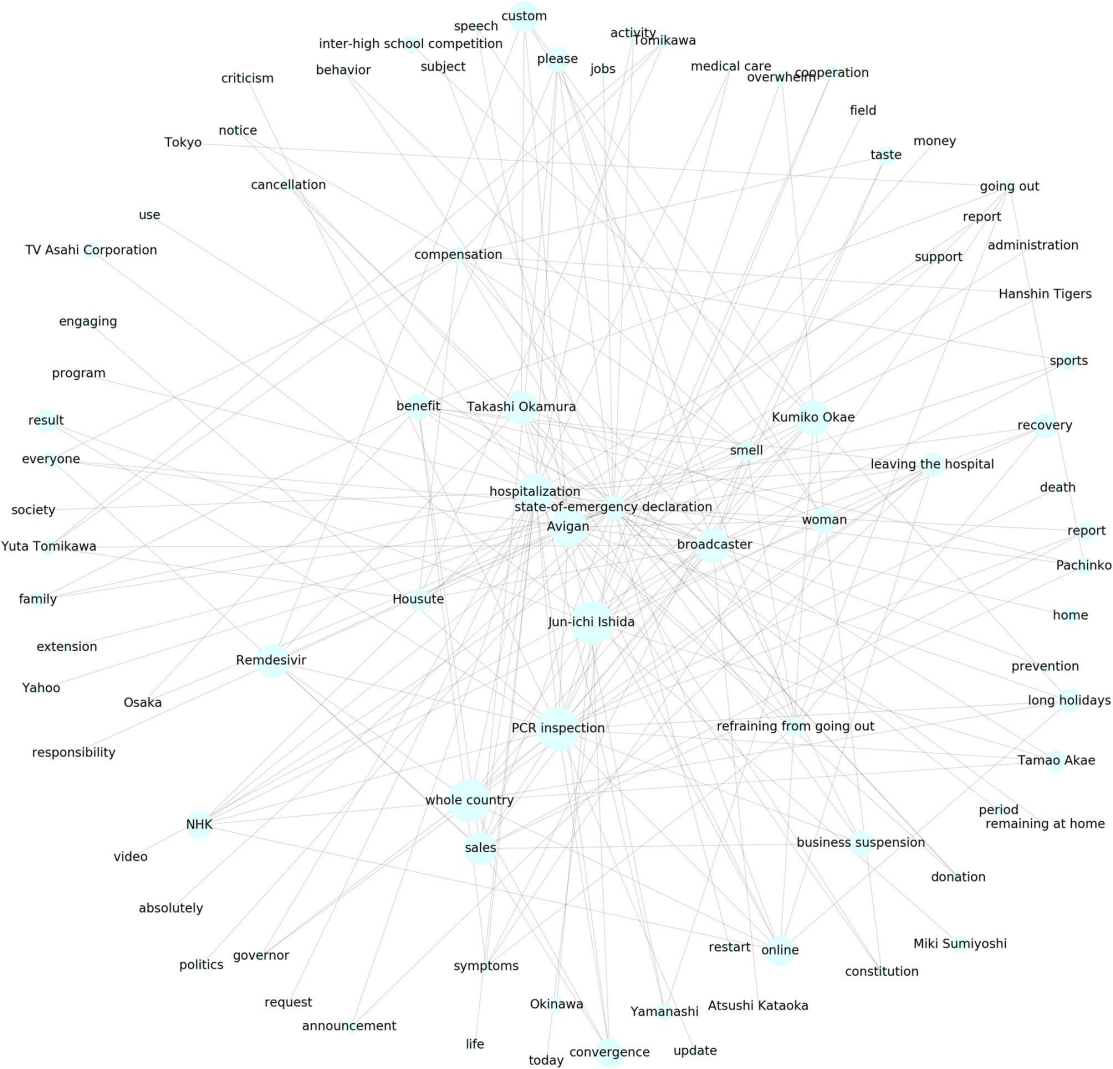
Second trend cluster in Fig. 5a. The words corresponding to each trend are shown.

Next, we show results of the proposed GLIPCA. We set the range of *B* to $\{1, 2, \ldots , 7\}$ and that of $\rho$ to $\{0.01, 0.015, \ldots , 0.05\}$. As described in “Methods”, GLIPCA uses the graphical lasso algorithm^22^ that estimates a sparse matrix with only direct correlations. Here, $\rho$ is a parameter that adjusts the strength of the sparsity. We then determined the *B* and $\rho$ that gave the best *Q*. Figures 5, 6 and 7 show the results of trend clustering. When compared with Fig. 1a, Fig. 5a reveals that GLIPCA achieved higher modularity than IPCA. This indicates that the graphical lasso algorithm is useful for extracting discriminative clusters. Specifically, clusters 1 and 2 represent early trends and late trends, respectively. Thus, we confirm that GLIPCA achieved (i). Moreover, Table 2(b) presents the results for the Shapiro-Wilk test. We confirm that GLIPCA achieved (ii) more successfully than IPCA. This indicates that GLIPCA detects not the usual trends based on seasonal events but unique trends caused by the influence of COVID-19. The graphical lasso algorithm assumes that each trend is generated from the unimodal Gaussian distribution. Therefore, trends with waveforms similar to such a distribution are likely to be detected. Note that it is clear that GLIPCA includes only direct correlations because the graphical lasso algorithm is adopted.

**Table 2:** Rates of trends within clusters, which are generated from the unimodal Gaussian distribution.

(a)				(b)		
	Cluster 1	Cluster 2	Cluster 3		Cluster 1	Cluster 2
$p\ge 10^{-2}$	0.0916	0.0625	0.000	$p \ge 10^{-2}$	0.156	0.172
$p\ge 10^{-3}$	0.146	0.125	0.000	$p \ge 10^{-3}$	0.300	0.333
$p \ge 10^{-4}$	0.172	0.156	0.000	$p \ge 10^{-4}$	0.356	0.448

We investigated whether the *p* values of the Shapiro–Wilk test for the trends are greater than the thresholds. (a) Results for IPCA. (b) Results for GLIPCA.

## Discussion

**Table 3:** Rates of early trend words and late trend words, as defined by Google Trends, within the clusters.

(a)				(b)		
	Cluster 1	Cluster 2	Cluster 3		Cluster 1	Cluster 2
Early trend words	0.600	0.563	0.706	Early trend words	0.889	0.368
Late trend words	0.400	0.438	0.294	Late trend words	0.111	0.632

(a) Results for IPCA. (b) Results for GLIPCA.

We quantitatively discuss the results for (i) in “Performance verification”. Concretely, we used Google Trends (https://trends.google.co.jp/trends/) to classify each word as an early or a late trend word. Table 3 lists the rates of early and late trend words within the obtained clusters. This table shows that GLIPCA extracts trends that correlate with trends defined by Google Trends, i.e., public opinions. This suggests that GLIPCA enables us to gain an overview of timely news about COVID-19 and would support decision-making based on data. Subsequently, we observe details of cluster 1 (early trends) and cluster 2 (late trends) obtained by GLIPCA.

First, we discuss cluster 1 obtained by GLIPCA, which is shown in Fig. 6. In cluster 1, the membership degrees of “events”, “Olympics”, and “five rings” are especially high (0.369, 0.247, and 0.242, respectively). The membership degrees are mathematically defined as each element of $\varvec{u}$ in (3), and represent degrees of each trend (word) belonging to the clusters. In Japanese, “Olympics” and “five rings” have the same meaning. These words may reflect the international argument about whether the Tokyo 2020 Summer Olympics should be held. Furthermore, we observed the domestic news about the death of Ken Shimura, a famous Japanese comedian who played the character “Bakatono”, on March 30, 2020. A report^31^ found that the number of people that refrained from commuting increased after this time (from March 30, 2020, to April 5, 2020).

Second, we discuss cluster 2 obtained by GLIPCA, which is shown in Fig. 7. In cluster 2, news related to the state-of-emergency declaration became prominent. Specifically, we observed requests for remaining at home and business suspension by the government. Similar to cluster 1, the infections and/or deaths of Japanese celebrities such as Kumiko Okae, Jun-ichi Ishida, Yuta Tomikawa, Tamao Akae, and Miki Sumiyoshi were observed. This suggests the tremendous social impact of the words and actions of such celebrities. In contrast to cluster 1, in cluster 2, medical and scientific topics about COVID-19 such as the polymerase chain reaction (PCR) test, Remdesivir drug, Avigan drug, and loss of taste and smell were observed. This may be because people’s attention moved from worry and fear about an unknown novel pneumonia to the necessity of medical care and a new lifestyle as well as the scientific characteristics of COVID-19.

We explain how to use separation of early and late trends by GLIPCA for real-world applications. This separation allows us to understand dominant words for a certain time period from a flood of information changing day by day. Here, GLIPCA can prevent miscellaneous words generated based on indirect correlations from confusing people. Moreover, membership degrees of each word to early and late trend clusters give us ranking of words by the importance. These advantages help us objectively understand changes of people’s needs, public opinions, and government policies over time. As a result, GLIPCA would give us understanding about surrounding situations and suggestions for future actions on the basis of data.

## Comparison between GLIPCA and existing time series clustering method

We discuss the applicability and advantages of GLIPCA by comparing GLIPCA with *k*-shape^32^. We notice that *k*-shape is the representative time series clustering method and widely used in latest studies^33–35^. GLIPCA’s strong ability to cluster certain data, i.e., to extract essential trends from miscellaneous tweets, is derived from the graphical lasso algorithm^22^. This can be applied to not only in the proposed GLIPCA but also in other time series clustering algorithms. The contributions of this study include showing the effectiveness of introducing the graphical lasso algorithm into trend clustering about COVID-19.

**Figure 8:**
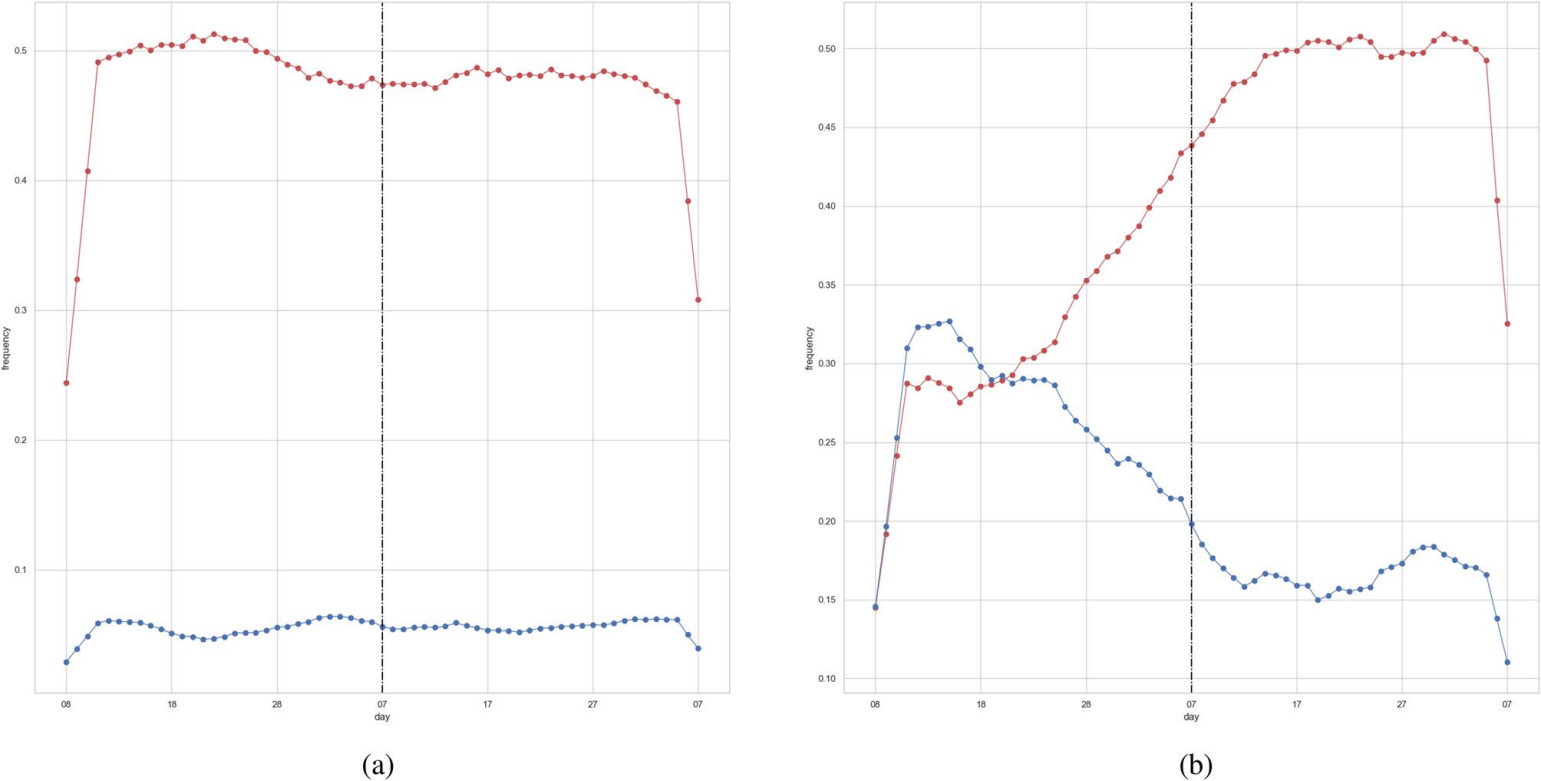
Time series signals for each cluster. The first and second trend clusters are denoted by red and blue, respectively. We show the averages of all trends in each cluster. The vertical line indicates the day of the state-of-emergency declaration (April 7, 2020). Although Tables 4 and 5 show the average and standard deviation over 10 trials, these figures show results for one trial. (**a**) Results for *k*-shape^32^. (**b**) Results for *k*-shape^32^ with the graphical lasso algorithm^22^.

We verify that the performance of *k*-shape^32^ is improved by adopting the graphical lasso algorithm. We applied *k*-shape to all trends including indirect correlations. In contrast, *k*-shape with the graphical lasso algorithm was applied to only trends with direct correlations. Such trends were calculated using the graphical lasso algorithm as in GLIPCA. Note that *k*-shape needs to manually determine the number of clusters. For fair comparison, we set the number of clusters to 2 as in GLIPCA. Similarly, we set *B* and $\rho$ to 6 and 0.015 as in GLIPCA. Table 4 shows that the graphical lasso algorithm improves the ability of modeling trends using the unimodal Gaussian distributions. Moreover, Fig. 8 and Table 5 indicate that the graphical lasso algorithm improves the ability of separating trends into early and late ones. Comparison with Tables 3 and 5 shows that GLIPCA separates early and late trends more successfully than *k*-shape with the graphical lasso algorithm. This may be because GLIPCA utilizes not only existence of direct correlations but also their strength, unlike *k*-shape with the graphical lasso algorithm.

Note that GLIPCA also has advantages over other time series clustering methods. Specifically, because our method results in an eigenvalue problem, the solution is uniquely determined. On the contrary, as we can see the standard deviation in Tables 4 and 5, other methods such as *k*-shape^32^ yield different results per trial because of initialization dependency. Our method also automatically determines the number of clusters. On the other hand, *k*-shape needs manual setting of the number of clusters. Although we could determine the number of clusters according to GLIPCA in this experiment, it is difficult to automatically determine the best number in general. These advantages of GLIPCA make parameter settings easy to determine, resulting in high reproducibility. Furthermore, it increases the interpretability or objectivity of trend clustering results for people’s decision-making.

**Table 4:** Rates of trends within clusters, which are generated from the unimodal Gaussian distribution.

(a)			(b)		
	Cluster 1	Cluster 2		Cluster 1	Cluster 2
$p \ge 10^{-2}$	$0.0569 \pm 0.0179$	$0.136 \pm 0.0387$	$p \ge 10^{-2}$	$0.195 \pm 0.0308$	$0.162 \pm 0.0234$
$p \ge 10^{-3}$	$0.0944 \pm 0.0275$	$0.249 \pm 0.0685$	$p \ge 10^{-3}$	$0.359 \pm 0.0475$	$0.313 \pm 0.0441$
$p \ge 10^{-4}$	$0.108 \pm 0.0330$	$0.315 \pm 0.0891$	$p \ge 10^{-4}$	$0.497 \pm 0.0710$	$0.367 \pm 0.0683$

We investigated whether the *p* values of the Shapiro–Wilk test for the trends are greater than the thresholds. The number of clusters and *B* were set to 2 and 6 as in the proposed GLIPCA. We performed experiments 10 times with different initialization values and show the average and standard deviation. (a) Results for *k*-shape^32^. (b) Results for *k*-shape^32^ with the graphical lasso algorithm^22^. $\rho$ was set to 0.015 as in GLIPCA.

**Table 5:** Rates of early trend words and late trend words, as defined by Google Trends, within the clusters.

(a)			(b)		
	Cluster 1	Cluster 2		Cluster 1	Cluster 2
Early trend words	$0.628 \pm 0.0807$	$0.551 \pm 0.120$	Early trend words	$0.448 \pm 0.0878$	$0.760 \pm 0.0525$
Late trend words	$0.372 \pm 0.0807$	$0.449 \pm 0.120$	Late trend words	$0.552 \pm 0.0878$	$0.240 \pm 0.0525$

Experimental conditions are the same as in Table 4. (a) Results for *k*-shape^32^. (b) Results for *k*-shape^32^ with the graphical lasso algorithm^22^.

**Figure 9:**
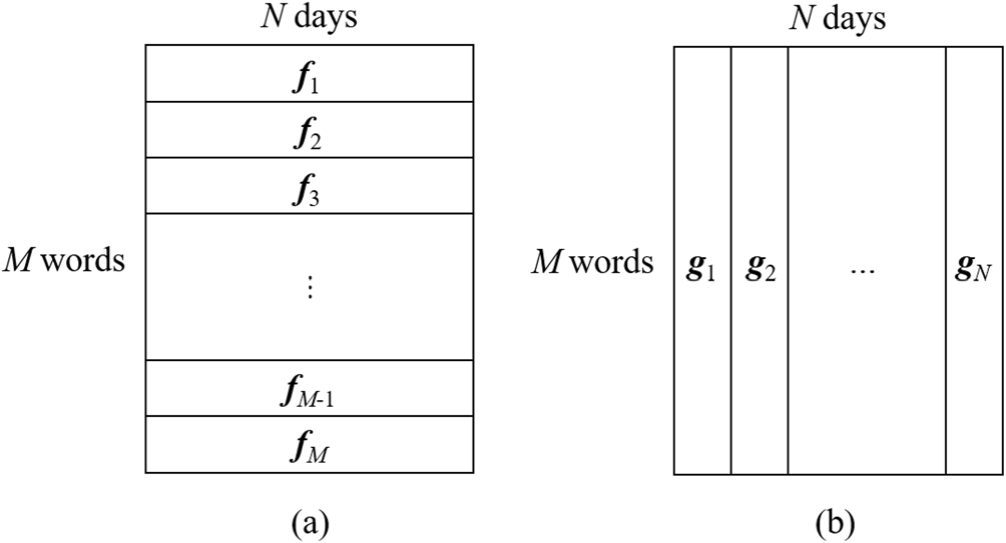
We observe the frequencies of *M* words for *N* days. (**a**) The frequencies of the *i*th word for *N* days are denoted by $\varvec{f}_i \in {\mathbb {R}}^{N}$ $(i=1, 2, \ldots , M)$. (**b**) For the *j*th day, the frequencies of *M* words are denoted by $\varvec{g}_j \in {\mathbb {R}}^{M}$ $(j=1, 2, \ldots , N)$.

## Methods

The proposed method for trend clustering using GLIPCA is explained. For each of the extracted *M* words (details are described in “Data used in this study”), we calculate a feature vector $\varvec{f}_i$ $\in {\mathbb {R}}^{N}$ $(i = 1, 2, \ldots , M)$. This feature vector aligns the daily frequencies of each word. Concretely, the *j*th element of $\varvec{f}_i$ represents the frequency of the *i*th word on the *j*th day. Note that the ranges of the word frequencies depend on each day. To normalize the ranges, we used the empirical distribution function^36^ on $\varvec{f}_i$. In this way, we regard the frequencies of each word included in the daily tweets as time series signals. Moreover, we apply a moving average filter with a width of *B* to $\varvec{f}_i$ for smoothing. Hereafter, we represent the observation as ${\mathscr {D}} = \{\varvec{g}_1, \varvec{g}_2, \ldots , \varvec{g}_N\}$ (see Fig. 9).

### Construction of the partial correlation network

The proposed method models essential trends as unimodal Gaussian distributions with direct correlations. To quantify the types of correlations between trends, we use the graphical lasso algorithm^22^. This algorithm enables the construction of a partial correlation network that connects only the words with direct correlations. Specifically, the graphical lasso algorithm performs a sparse estimation of the precision matrix $\varvec{\Sigma }^{-1}$ of ${\mathscr {D}}$ using the sample covariance matrix $\varvec{S}$ as follows:

1 $$\begin{aligned} \hat{\varvec{\Sigma }}^{-1} = \mathop {\mathrm{arg~max}}\limits _{\varvec{\Sigma }^{-1}} \{\ln \det \varvec{\Sigma }^{-1} -\mathrm {tr}(\varvec{S \Sigma }^{-1})-\rho \Vert \varvec{\Sigma }^{-1}\Vert _1\}. \end{aligned}$$

For optimization, we use a block coordinate descent method^37^. The estimated precision matrix $\hat{\varvec{\Sigma }}^{-1}$ is a sparse matrix with only direct correlations. The partial correlation matrix $\varvec{L}=(l_{ij})$ $(i= 1, 2, \ldots , M,\ j = 1, 2, \ldots , M)$ can be obtained as

2 $$\begin{aligned} l_{ij} = - \frac{\hat{ \varvec{\Sigma }}^{-1}_{i,j}}{\sqrt{\hat{\varvec{\Sigma }}^{-1}_{i,i} \hat{\varvec{\Sigma }}^{-1}_{j,j}}}, \end{aligned}$$

where $\hat{\varvec{\Sigma }}^{-1}_{i,j}$ denotes the (*i*, *j*)-th element of $\hat{\varvec{\Sigma }}^{-1}$.

The partial correlation matrix $\varvec{L}$ can be considered as a network whose nodes are trends (words). Edges are undirected and have weights that are equivalent to the strengths of the direct correlations. Therefore, we refer to $\varvec{L}$ as a partial correlation network hereafter. We also consider trend clustering as community detection in the partial correlation network.

In the graphical lasso algorithm in GLIPCA, we assume that ${\mathscr {D}}$ is generated from a multivariate Gaussian distribution. Therefore, it is assumed that each $\varvec{f}_i$ $(i=1, 2, \ldots , M)$ follows a unimodal Gaussian distribution. Under this assumption, pairs with similar waveforms are connected by edges. In other words, we model the target trends as ones generated by unimodal Gaussian distributions and remove those trends that follow other distributions from the subsequent processing.

### Trend clustering via GLIPCA

When using our method for decision-making to fight COVID-19, the clustering results should be stable and interpretable. To this aim, we adopt the HITS algorithm^23^ as the base of the proposed method. The HITS algorithm is a representative community detection method. In fact, it is equivalent to PCA for a network structure^24^. Because its solution is uniquely determined, this algorithm is suitable for our aim. Specifically, the HITS algorithm calculates the eigenvectors of $\varvec{L}^\mathrm {T} \varvec{L}$. The *i*th eigenvector corresponds to the *i*th community in the partial correlation network. More specifically, the *j*th element of the *i*th eigenvector represents the degree to which the *j*th trend (word) belongs to the *i*th community (cluster) in the network.

Here, we derive GLIPCA, i.e., the improved variant of the HITS algorithm. The HITS algorithm extracts orthogonal bases but generates duplicated members of different clusters. This makes it difficult for us to interpret the meaning of each cluster. To overcome this difficulty, GLIPCA first extracts dense nodes corresponding to the first principal component. Concretely, we calculate the $\varvec{u} = [u_1, u_2, \ldots , u_{M}]^\mathrm {T}$ that satisfies

3 $$\begin{aligned} \varvec{L}^{\mathrm {T}} \varvec{L} \varvec{u} = \lambda \varvec{u}, \end{aligned}$$

where $\lambda$ is the first principal component and $\varvec{u}$ is the corresponding eigenvector. Here, $u_i$ represents the membership degree of the *i*th trend (word) to the cluster corresponding to the first eigenvetcor. We can obtain the trend clustering result as the set of words

4 $$\begin{aligned} C = \{ i \ | \ u_i > Th \}. \end{aligned}$$

Here, *Th* is a threshold to determine the cluster members and was set to 0 in “Results”. If |*C*| $(|\cdot |$ denotes the number of elements in the set) is less than $Th_m$, this indicates that meaningful clusters are no longer obtained. In “Results”, $Th_m$ was set to 10. If $C < Th_m$, we do not perform the subsequent processing.

If $C \ge Th_m$, GLIPCA reconstructs the partial correlation network so that the extracted nodes can be removed. Concretely, we update network ***L*** as follows:

5 $$\begin{aligned} l_{ij} \leftarrow 0, \end{aligned}$$

where $l_{ij}$ is the (*i*, *j*)-th element of $\varvec{L}$ corresponding to the extracted nodes. Then, we obtain the subsequent cluster (set of trends) by performing (3) and (4). We note that the updated network does not include the words obtained as the previous cluster. By iteratively performing these procedures, we can obtain unduplicated clusters, unlike the original HITS algorithm (PCA).

It is difficult to quantitatively evaluate the accuracy of trend clustering results. However, the fact that GLIPCA generates clusters without duplicated members can overcome this difficulty. Because GLIPCA is formulated as flat community detection in a network, a quality measure called modularity^25^ can be used. The modularity *Q* is defined as

6 $$\begin{aligned} Q = \frac{1}{2 m} \sum _{i=1}^{M} \sum _{j=1}^{M} (w_{ij} - \frac{k_i k_j}{2 m}) \delta _{ij}, \end{aligned}$$

where

7 $$\begin{aligned} 2m = \sum _{i=1}^{M} \sum _{j=1}^{M} w_{ij}, \ \ \ k_i = \sum _{j=1}^{M} w_{ij}. \end{aligned}$$

Here, $\delta _{ij}$ is 1 if the nodes corresponding to $\varvec{f}_i$ and $\varvec{f}_j$ belong to the same cluster, and 0 otherwise. Moreover, $w_{ij}$ is 1 if an edge between nodes corresponding to $\varvec{f}_i$ and $\varvec{f}_j$ in the partial correlation network exists, and 0 otherwise. The use of *Q* enables us to determine the best parameters of $\rho$ and *B*. By searching for the values of $\rho$ and *B* that yield the highest *Q*, trend clusters are uniquely determined.

### IPCA algorithm

Finally, we explain IPCA, i.e., a reference method used in “Results”. IPCA is GLIPCA without the graphical lasso algorithm^22^, as described in “Performance verification”. Specifically, IPCA calculates the *k*-nearest neighbor network^38^ for trends $\{ \varvec{f}_1, \varvec{f}_2, \ldots , \varvec{f}_M \}$. Concretely, for each trend $\varvec{f}_i$ $(i=1,2, \ldots , M)$, we select *k* trends from $\varvec{f}_j$ $(j=1,2, \ldots , M, i \ne j)$ in descending order of cosine similarities to construct an unweighted network. We set *k* to 3 as in the study^27^. Note that the calculated network includes indirect correlations. Moreover, IPCA applies procedures shown in “Trend clustering via GLIPCA” to the calculated network for trend clustering. On the contrary, GLIPCA applies the same procedures to the partial correlation network $\varvec{L}$ with only direct correlations, as described above.

## Data Availability

The datasets generated and/or analyzed during the current study are available in the Twitter repository, https://twitter.com/, by performing crawling with the method shown in “Data used in this study”.
